# Promising Hybrid Graphene-Silver Nanowire Composite Electrode for Flexible Organic Light-Emitting Diodes

**DOI:** 10.1038/s41598-019-54424-3

**Published:** 2019-11-29

**Authors:** Huiying Li, Yunfei Liu, Anyang Su, Jintao Wang, Yu Duan

**Affiliations:** 1 College of Computer Science and Technology, Symbol Computation and Knowledge Engineer of Ministry of Education, Jilin University, Changchun, 130012 China; 2 College of Software, Jilin University, Changchun, 130012 China; 3 State Key Laboratory of Integrated Optoelectronics, College of Electronic Science and Engineering, Jilin University, Changchun, 130012 China

**Keywords:** Graphene, Nanowires

## Abstract

Thanks to its high transparency, high carrier mobility, and thermal conductivity, graphene is often used as transparent conductive electrode (TCE) in optoelectronic devices. However, the low carrier concentration and high resistance caused by vacancy defects, grain boundaries, and superposed folds in typical graphene films limit its application. In this study, we propose a method to increase both the conductivity and carrier concentration in single-layer graphene (SLG) by blending it with silver nanowires (AgNWs). AgNWs provide connections between grain boundaries of graphene to improve charge-carrier transport. The AgNWs in this study can reduce the resistance of SLG from 650 Ω/◻ to 27 Ω/◻ yet still maintain a transmittance of 86.7% (at 550 nm). Flexible organic light-emitting diode, with a maximum 15000 cd m^−2^ luminance was successfully fabricated using such graphene and AgNWs composite transparent electrodes.

## Introduction

Transparent conductive electrodes (TCEs) are important components in optoelectronic devices, such as touch-screen displays^1^, photoelectric sensor^2^, organic photovoltaics (OPVs)^3–5^, and organic light-emitting diodes (OLEDs)^6–8^. Indium tin oxide (ITO) has been the most popular TCE for decades because of its excellent photoelectric properties. However, with the development of science and technology and increasing human needs, the trend for future electronic equipment is to became flexible, wearable and manufactured at low cost^9–11^. Because ITO is subject to potential shortages of indium and tends to be brittle i.e. non-flexible, there is a need for suitable alternative transparent electrode materials. In the past, the potential candidate materials such as metallic nanowires^12,13^, conductive polymers^14^, carbon nanotubes^15,16^ and graphene^17,18^ have been investigated. Among these, single-layer graphene (SLG), with its extremely high carrier mobility (>10^3^ cm^2^·V^−1^·s^−1^) and transmittance (97%), represents perhaps the most promising alternative. However, large-area growth and transfer of graphene is difficult to accomplish efficiently. Many defects can occur, such as point defects, impurity defects on graphene sheets^19,20^ and grain boundaries^21^. Moreover, with massive boundaries, a single graphene is isolated into nanosheets. These defects and overlaps between graphene sheets can cause high resistance in SLG films. In order to improve its conductivity, several procedures, like stacking^22^, acidification^23^, chemical doping^24^ and physical mixing with other conductive materials^25^, were studied in the past. *Han et al*. successfully fabricated efficient white OLEDs using a four-layer stacked graphene anode, respectively, which was doped with AuCl_3_ and HNO_3_^6^. *Zhang et al*. modified SLG with Al-TiO_2_ and used graphene/Al-TiO_2_ composite film as the cathode in organic solar cells^26^. *Seo et al*. used graphene-silver nanowire (AgNWs) hybrid structure as electrode in ultraviolet light emitting diodes^27^. *Xu et al*. used graphene–AgNWs hybrid films as electrodes for loudspeakers^28^. However, they paid more attention to the application of this structure, and did not deeply explore the interaction between the AgNWs and graphene and the principle of AgNWs to improve the conductivity of graphene. *Dong et al*. also studied the graphene-AgNWs structure and applied structure to OLED^29^. However, they also did not study the mechanism in depth, and their device performance had much room for improvement, the luminance was below 5000 cd m^−2^ at 13 V.

In our work, we blend graphene with AgNWs to take advantage of beneficial composite structures of one-dimensional and two-dimensional materials to improve the electrical properties of graphene. AgNWs with very high length-diameter ratios were used to ensure a good combination of optical and electrical properties. The primary purpose of the nanowires is to reduce the effect of grain boundaries and overlap defects between graphene sheets by providing additional conductive pathways. Simultaneously, the carrier concentration of the SLG film can be increased. We also studied the interaction mechanism between graphene and AgNWs and considered the percolation theory of binary systems. To ensure suitability for optoelectronic devices, several flexible, green, organic light-emitting devices were fabricated with this novel transparent and flexible composite electrode. These devices reached a luminance of over 15000 cd m^−2^.

## Results

**Figure 1:**
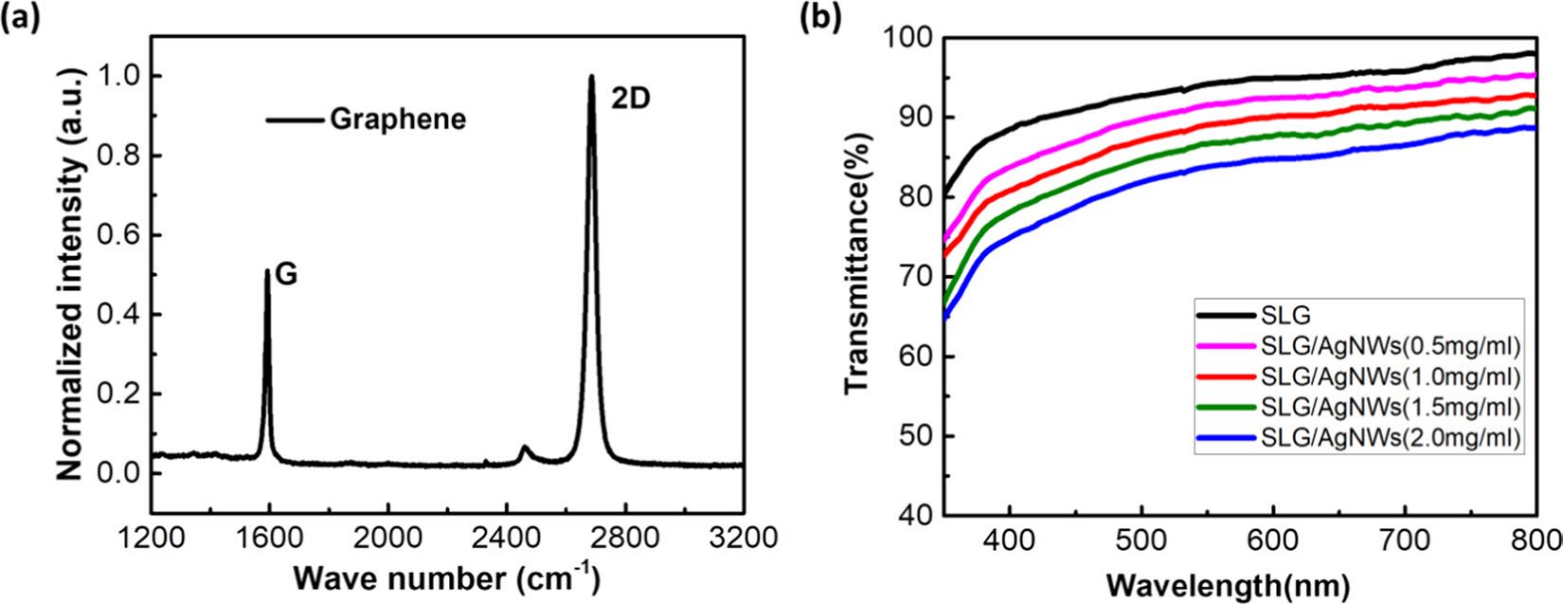
(**a**) The Raman spectra of graphene. (**b**) Optical transmittance spectra of the graphene film and the SLG/AgNWs composite films with different AgNWs concentrations.

Prior to fabricate the composite electrode, we first studied the graphene film via Raman spectroscopy. Figure 1(a) shows the Raman spectra of the graphene we used. The spectra show a typical peak distribution: D, G and 2D bands with the corresponding wave numbers of 1356 cm^−1^, 1592 cm^−1^ and 2684 cm^−1^. The G peak is characteristic main peak of graphene, which is caused by the in-plane vibration of carbon atoms (sp^2^). The 2D peak is the two-phonon Raman peak, which is used to characterize the stacking mode of carbon atoms in graphene samples. The D peak is commonly seen when defects are present in carbon materials. The normalized intensity ratio of the D and G peaks (I_D_/I_G_) can be used as an indicator of defect quantity. The normalized intensity-ratio of the 2D and G peaks (I_2D_/I_G_) can identify single-layer graphene (SLG). Figure 1(a) shows a low I_D_/I_G_, which means there are not many defects, and the I_2D_/I_G_ ≈ 2 means the graphene we used was SLG^30^.

Optical transmittance is a key property of transparent electrodes. Transmittance spectra of SLG and SLG/AgNWs composite films with different AgNWs concentrations are shown in Fig. 1(b). Due to the surface morphology and the metallic properties of AgNWs, surface plasmon effect could take place on the metal/organics interface. Moreover, due to the strong optical reflection of metal, significant microcavity effect can be observed in the devices fabricated with high-density-AgNW layer. Therefore, the optical transmittance of the SLG films is 94.2% at 550 nm, and for SLG/AgNWs composite film with concentration of 0.5 mg/ml the transmittance is 91.5%. When the concentration increases from 0.5 mg/ml to 1.5 mg/ml, the transmittance of composite film can remain at 86.7%, which is higher than for commercial ITO (86%). When at high concentrations (2.0 mg/ml), the transmittance can still reach 83.8% at 550 nm. Moreover, the SLG/AgNWs composite layer showed same trend in spectra while holding high transmittance.

**Figure 2:**
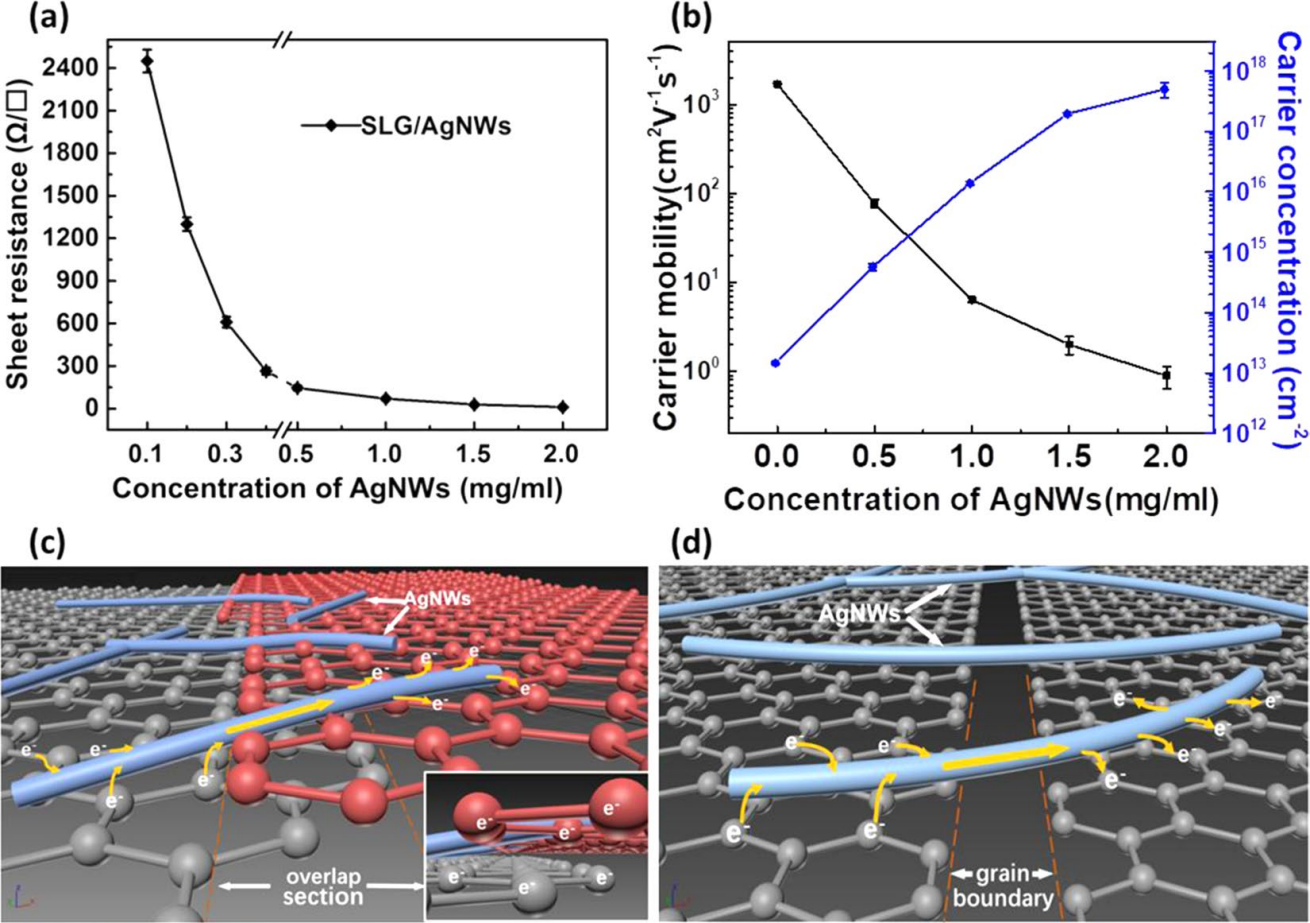
(**a**) Sheet resistance of SLG/AgNWs composite films with different concentrations of AgNWs. The schematic illustrates the possibilities of the AgNWs to reduce the resistance of graphene. (**b**) Carrier mobility and carrier concentration of SLG/AgNWs composite films for different concentrations of AgNWs. (**c**) AgNWs provide a more accessible path for electrons to move than hoping between graphene layers within the overlapping sections. The insert shows a front view of electron transport within an overlapping section. (**d**) AgNWs provide new conductive “bridges” between the grain boundaries of graphene.

To investigate the conductive properties of the composite electrode, the sheet resistance (R_s_) of the sample films was tested with a four-point probe instrument. The R_s_ of SLG/AgNWs composite films with different concentrations of AgNWs (0.1, 0.2, 0.3, 0.4, 0.5, 1.0, 1.5, 2.0 mg/mL, and 0 mg/ml i.e. SLG-only) are shown in Fig. 2(a). The R_s_ of the as-prepared SLG can reach up to ~650 Ω/▯, as a result of a mass production size (21 cm × 29 cm). The high sheet resistance of SLG could be result from the two different types of defects: The first type of defects occurs on the surface of graphene sheets, such as dots and single vacancy defects. The electron-scattering centers form on the surface of graphene because of these defects. They cause electron scattering, affect the electron transfer, and, ultimately, decrease the conductivity of graphene^31^. The second type of defects occurs between graphene sheets. They are caused by wrinkles, grain boundary, overlap sections. Large-area graphene-growth in different regions does not guarantee a uniform extension of the crystal orientation. When the regional graphene grows to a certain size, cross-regions begin to appear, and different crystal orientations lead to the formation of graphene line defect, wrinkles, and overlapping stacks^32^. The above mentioned first defect needs mainly chemical modification. In this work, we used AgNWs to reduce Rs of SLG to create more accessible conductive pathway, which can solve the effects of second type.

Figure 2(a) shows the curves of R_s_ with different concentration of AgNWs. When the AgNWs concentration is below than 0.3 mg/ml, the value of R_s_ of the composite film exceeds that of pure graphene. Therefore, there are two possibilities: (I) When the AgNWs do not form a two-dimensional film, the introduction of a very small number of AgNWs destroys the planar structure, which is equivalent to introducing impurity defects. (II) When the AgNWs have just formed the two-dimensional film, the density of AgNWs is not efficient to connect from each other, leading to little improvement compared with the contact resistance between SLG and AgNWs.

**Figure 3:**
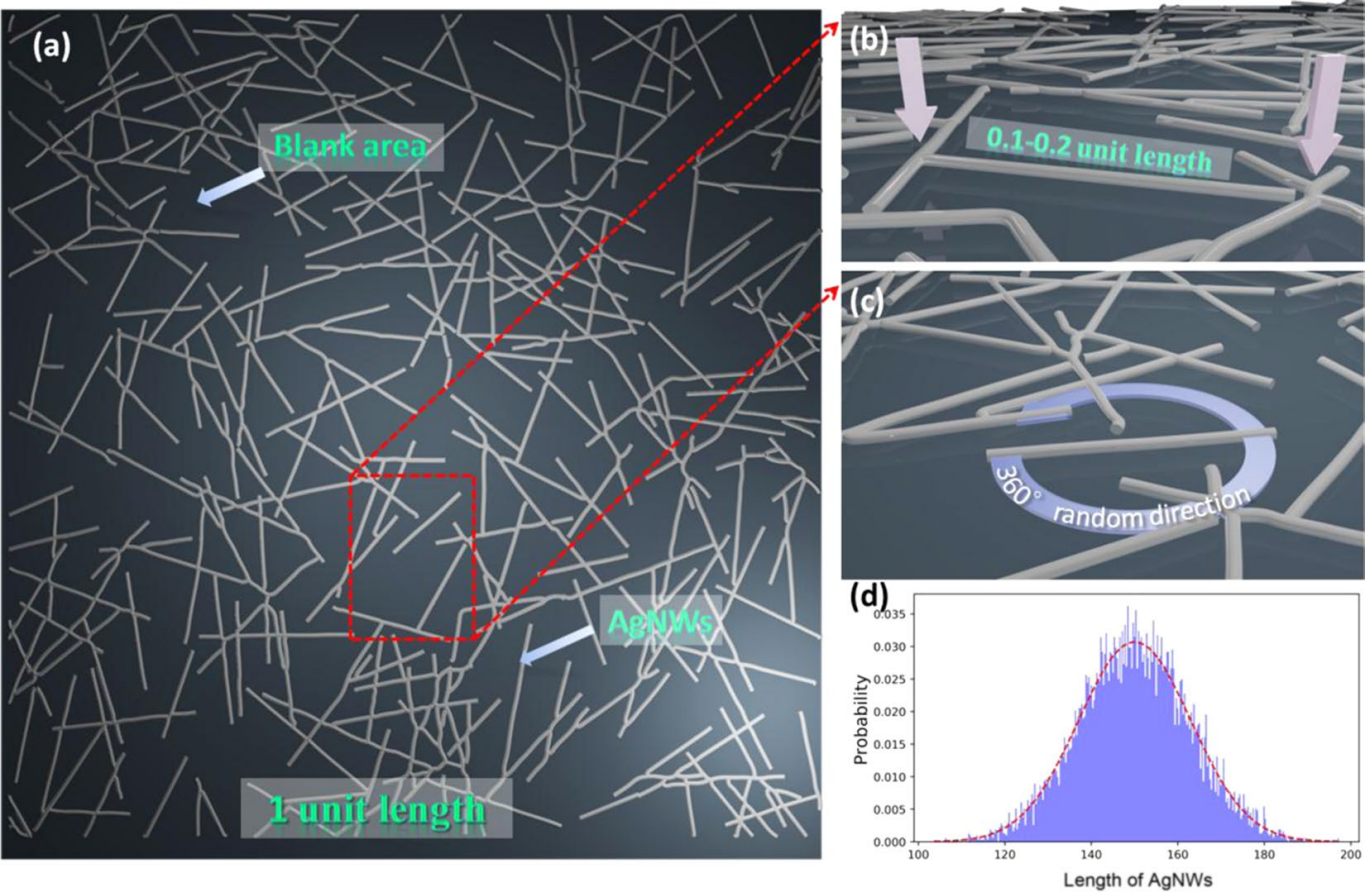
The simulated diagram of AgNWs network at the percolation concentration used by Monte Carlo method. (**a**) The whole plane of AgNW network during one singe simulation process. The AgNWs were varied in (**b**) length and (**c**) angles. (**d**) Probability of AgNWs in versus of their length. The normal distributions were accorded to the actual distributions of synthesized AgNWs.

To investigate this critical factor, we conducted a simulation of the percolation using the Monte Carlo method^33,34^. Herein, AgNWs are simulated as thin rods with negligible diameter, and the rod length is in the range of 0.1~0.2 unit length, shown in Fig. 3(b). Moreover, the direction of AgNWs is randomly distributed in the horizontal plane of electrode as shown in Fig. 3(c). To approximate realistic parameters, the selection of length obeys normal distribution, shown in Fig. 3(d). A Monte Carlo random number method was used to generate the midpoint of a thin rod, and a geometrical relation is used to judge whether the rods intersect. For the unit area, if the arbitrary sides are allowed to pass, it was assumed that a conducting network was formed. By updating an IDs array, we can judge whether the unit area is connected. According to these conditions, the concentration of the AgNWs, which corresponds to the minimum number of thin rods, is the critical parameter. It is defined as percolation concentration (*Cp*). We calculated that the percolation concentration is about 0.1 mg/ml, and the error is less than 5%. The simulated diagram of AgNWs at the percolation concentration used Monte Carlo method is shown in Fig. 3(a).

**Figure 4:**
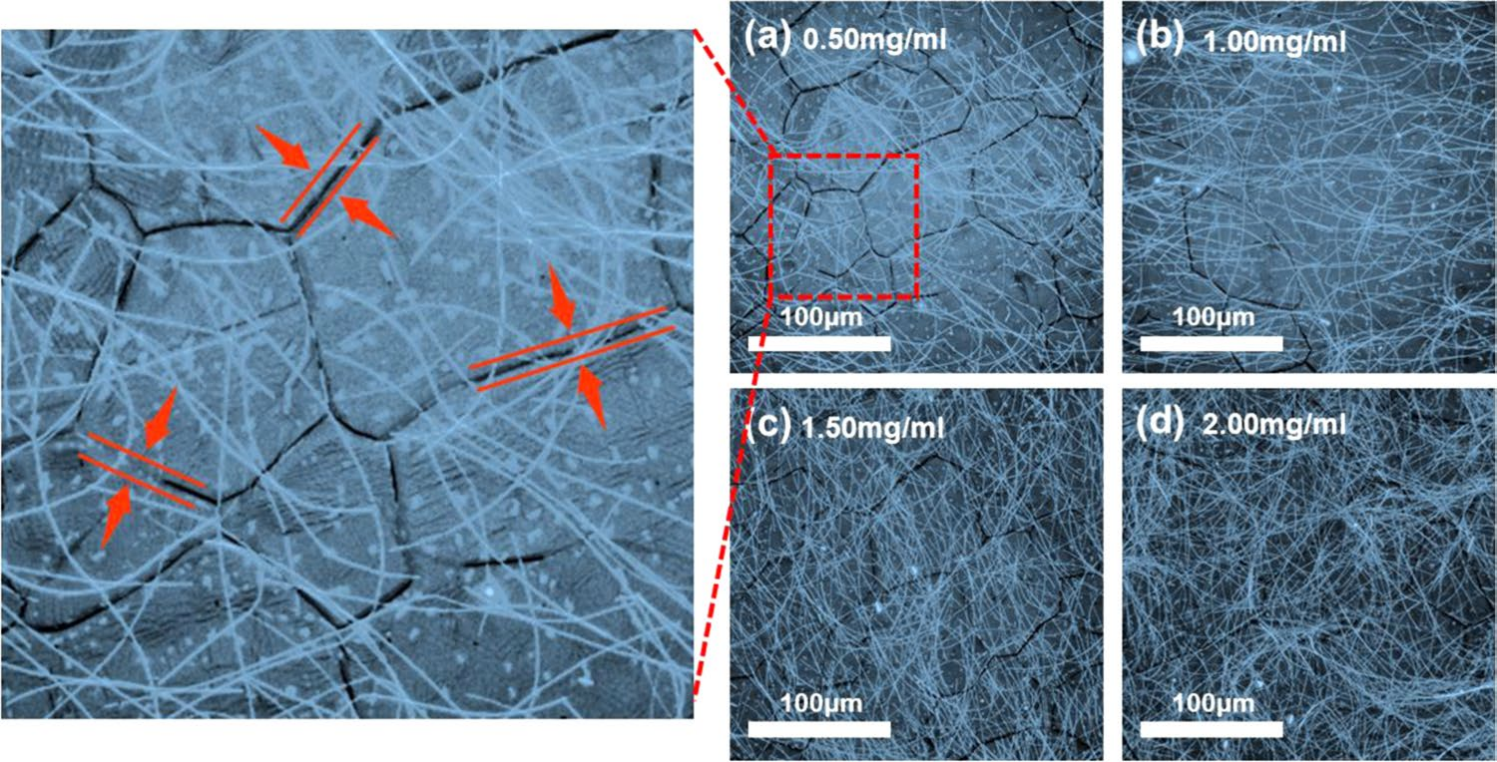
The 3D Laser Scanning Microscope images of SLG/AgNWs composite film with (**a**) 0.5 mg/ml; (**b**) 1.0 mg/ml; (**c**) 1.5 mg/ml; and (**d**) 2.0 mg/ml concentration of AgNWs. High power microscope images of grain boundaries of SLG.

At 0.3 mg/ml, the negative effects caused by the contact resistance and the positive “bridge effect” are just offset. The positive “bridge effect” is illustrated in Fig. 2(c,d). The figures also explain how the AgNWs reduce the R_s_ of graphene. Between overlapping sections of graphene, the carrier transport is blocked. Simulation results showed that AgNWs could effectively percolate on SLG in concentrations over 0.3 mg/ml, indicating that most graphene nanosheets were connected by AgNWs. AgNWs provide a better-accessible pathway for electron transfer than inter-layer hopping between graphene sheets - see Fig. 2(c). There was no efficient conducting path for carrier transport at the grain boundaries of the graphene sheet. The AgNWs, however, provided new conductive “bridges” between grain boundaries of graphene – see Fig. 2(d), which can also be seen from the enlarged drawing in Fig. 4. These are the two main-mechanisms how AgNWs reduce the defect effects between graphene sheets, and thereby also reduce the Rs of the composite electrode.

R_s_ decreased to ~270 Ω/▯ for 0.5 mg/ml and reached ~27 Ω/▯ at concentration of 1.5 mg/ml. Even though the concentration of the AgNWs was over 1.5 mg/mL, the R_s_ improved only slightly, ~15 Ω/▯ at concentration of 2.0 mg/ml, this is because AgNWs have formed a dominant and independent conducting network.

The experimental data are also in agreement with percolation theory for single material systems^35–37^, which follows an inverse scaling function for electrical conductivity:

$$\frac{1}{{R}_{s}} \sim {(C-{C}_{p})}^{k}$$

here, *R*_*s*_ denotes the sheet resistance of the composite film. *C* denotes the concentration of the AgNWs. *C*_*p*_ denotes percolation concentration and *k* denotes the conductivity exponent, whose value ranges from 1~2 for two-dimensions. The formula is also applicable for composite electrode systems, and hence this study. This provided the theory supports to the ratio of length to diameter of the selected AgNWs (70 nm × 100 um).

We also measured both carrier mobility and concentration of SLG/AgNWs composite films using a Hall-effect measurement system. The carrier mobility decreased sharply with increasing concentration, while the carrier concentration increased by an order of magnitude - see Fig. 2(b). For intrinsic graphene, the carrier mobility could reach ~1700 cm^2^·V^−1^·s^−1^, yet the carrier concentration was very low, about 1.5 × 10^13^cm^−2^. A low carrier concentration limits hole-injection, which causes unbalanced electron-hole recombination in the device. Figure 2(b) shows that the carrier concentration of the composite electrode increased to (5.7 ± 0.6) × 10^14^cm^−2^ for an AgNWs concentration of 0.5 mg/ml. When the AgNWs concentration reached 2.0 mg/ml, the carrier concentration was as high as (5.0 ± 0.9) × 10^17^cm^−2^. This increasing trend, however, soon slowed down. The reason for this is that the AgNWs formed a separate conducting network whose carrier concentration approaches the upper limit. The 3D laser scanning microscope images in Fig. 4 show the formation of AgNWs network on graphene films. With the increase of concentration, the network of AgNWs became more and more dense. Finally, in Fig. 4(d), the graphene layer has not even been seen. This loss of the significance of modified graphene, so when prepared the device, this concentration of electrode sample was not selected.

**Figure 5:**
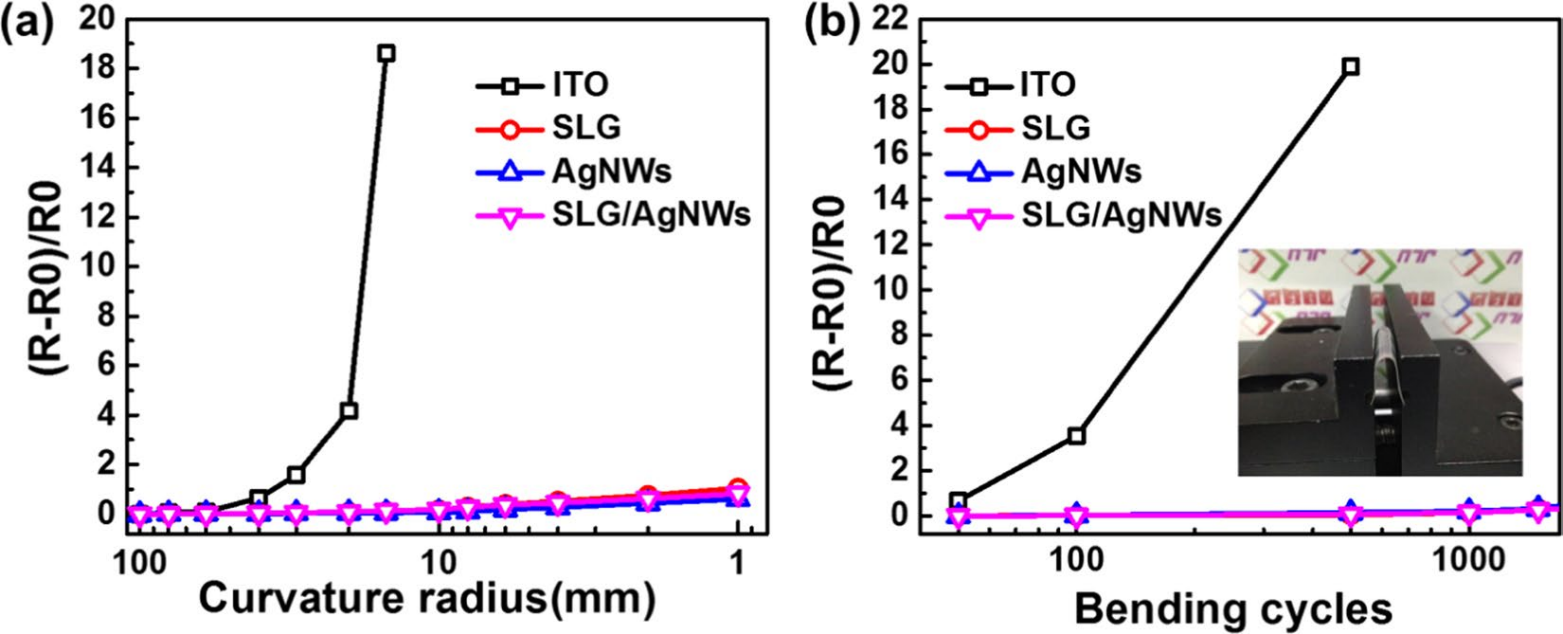
Details of the bending test of the ITO electrode on the PET substrate, SLG, AgNWs, and the SLG/AgNWs (1.5 mg/ml) composite electrode on the NOA63 substrate. (**a**) Curvature radius test results of the different electrodes after 100 bending cycles. (**b**) Bending cycle test results of the different electrodes for a curvature radius of 20 mm. The insert shows a picture of a SLG/AgNWs composite film on NOA63 substrate during bending in the test setup.

Figure 5 shows the bending test results for ITO electrode on a PET substrate, SLG, AgNWs and a SLG/AgNWs composite electrode on the NOA63 substrate. The relative change rate (R − R_0_)/R_0_ was used to compare the flexibility data of these test samples. ITO showed a sharp increase in Rs when the curvature radius was less than 20 mm, see Fig. 5(a), or the bending cycle number exceeded 100, see Fig. 5(b). The respective numbers for the three other samples remained almost constant. The composite film showed high mechanical robustness compared to the SLG or AgNWs-only film, because the components complement each other even at the curvature radius of 1 mm.The reasons for this are: Both graphene and the AgNWs are flexible materials with excellent mechanical stability. In addition, the stripping procedure needed to embed the electrode in the curing glue to tackle the rough surface, also improves the adhesion of the electrode. It can also provide mechanical protection for electrodes.

**Figure 6:**
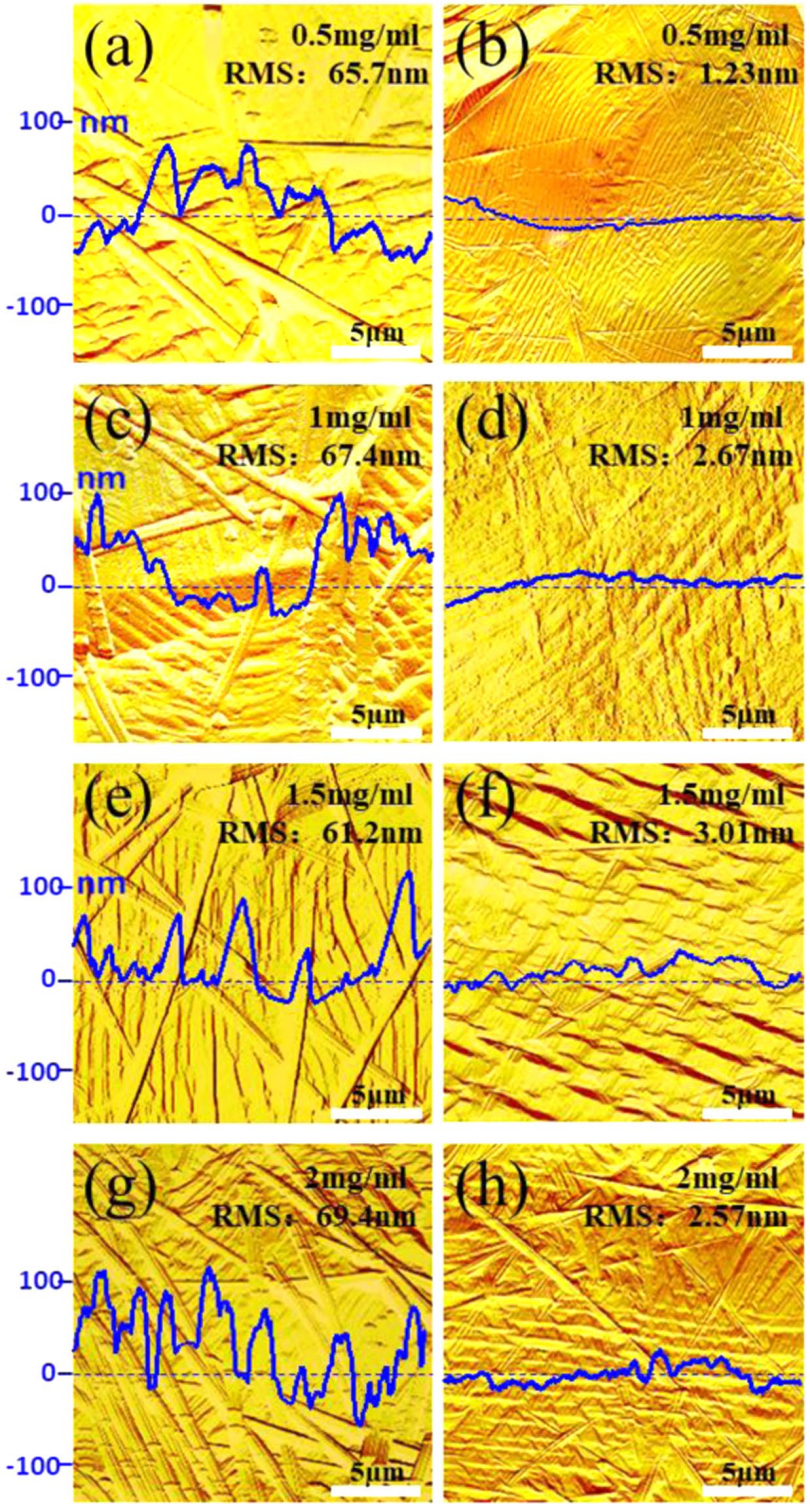
The AFM pictures of SLG/AgNWs composite electrodes with different concentrations AgNWs (0.5, 1.0,1.5, 2.0 mg/ml) before and after substrate transfer (**a**,**c**,**e**,**g** are on PET and **b**,**d**,**f**,**h** are on NOA63).

After measuring and analyzing the electro-optical and mechanical properties of composite electrodes, it is necessary to analyze the surface roughness of composite electrodes. We used AFM to test the surface morphology of four different composite electrodes. Figure 6 shows the AFM Amplitude error graphs of SLG/AgNWs composite electrodes with different concentrations AgNWs (0.5, 1.0, 1.5, 2.0 mg/ml) before and after substrate transfer. Figure 6(a,c,e,g) are the composite electrodes on PET, and Fig. 6(b,d,f,h) are on NOA63. From the contour maps on both sides, the decrease in the fluctuation is obvious. Before transfer, the naked AgNWs could be clearly observed, and the RMS of composite electrodes are about 60 nm~70 nm. After transferring electrode with NOA63, AgNWs were embedded in NOA63, and the RMS reduced to 1 nm~3 nm, which was an obvious improvement. The transfer technology solved the problems of loose and easily aggregate of AgNWs, which is more consistent with the requirements of the OLED electrode.

**Figure 7:**
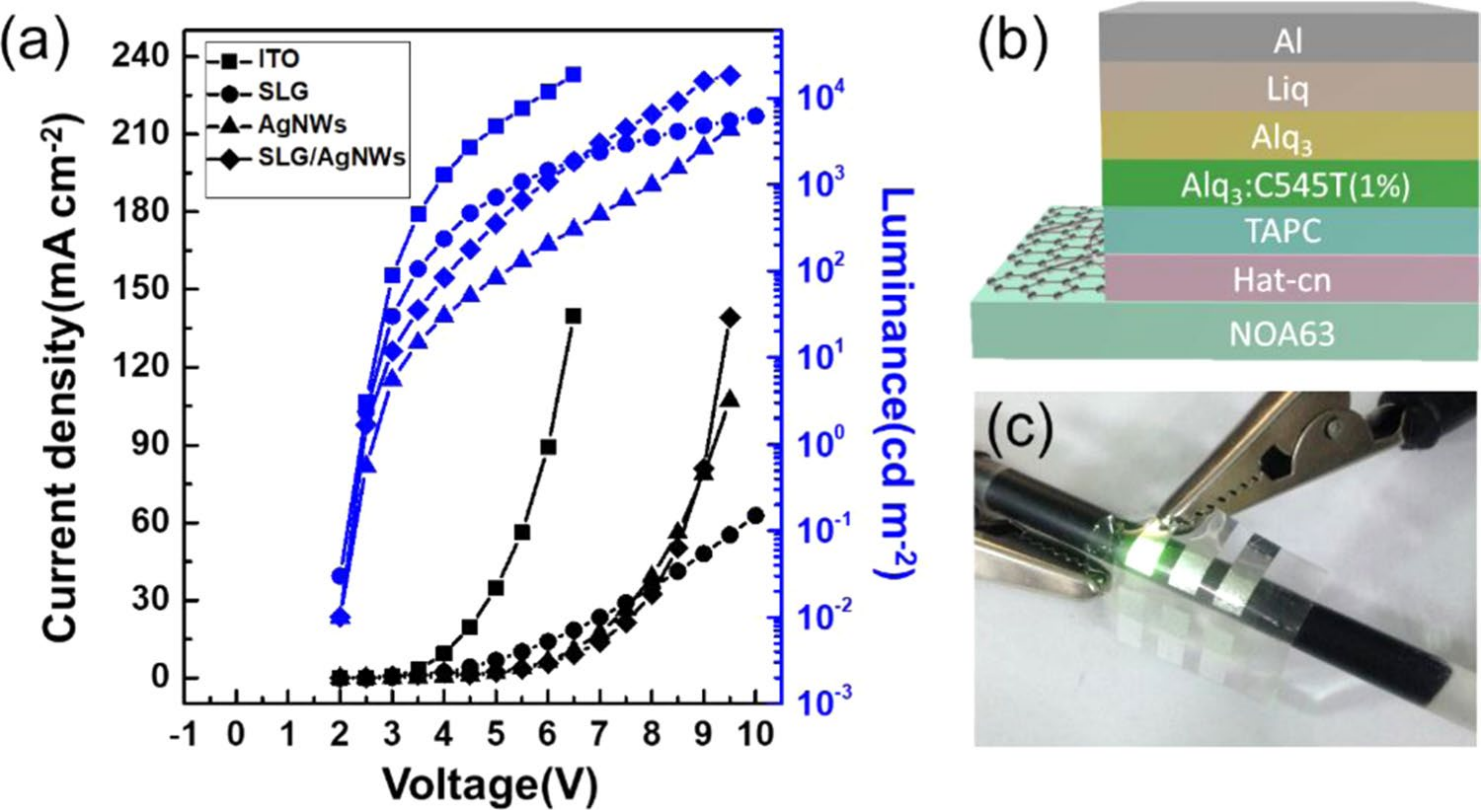
(**a**) J-V-L curve and current efficiency of OLED devices with the ITO, SLG, AgNWs and SLG/AgNWs composite electrodes. (**b**) Schematic illustration of the green OLED structure. (**c**) Photographs of the flexible green OLED with the SLG/AgNWs composite electrode.

**Figure 8:**
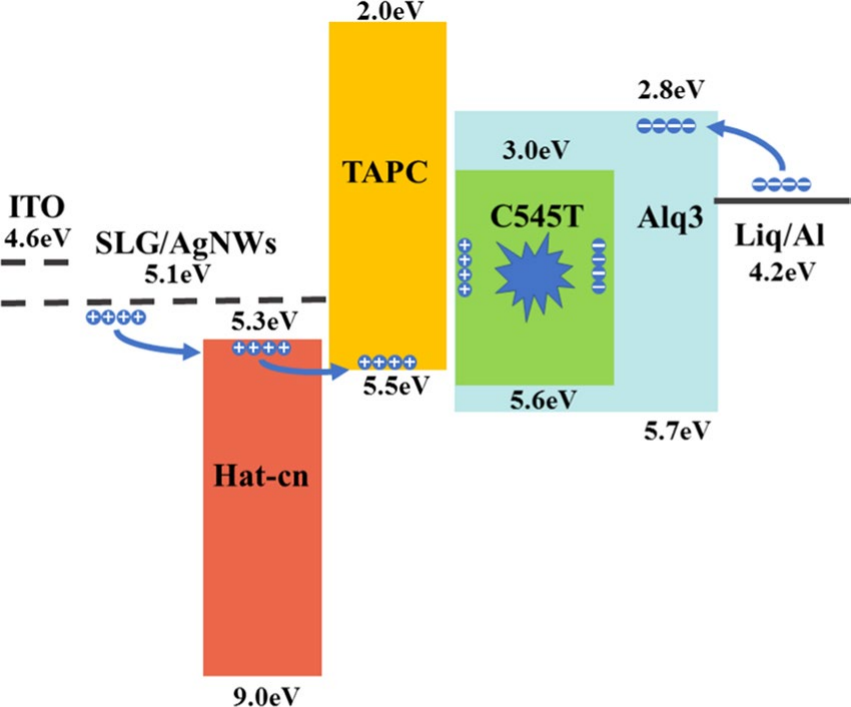
The energy level diagrams of organic light-emitting devices with ITO or SLG/AgNWs composite electrodes.

Considering that a high concentration of AgNWs film might negatively affect both optical transparency and surface roughness of the electrode, we chose composite electrode with a 1.5 mg/ml concentrations of AgNWs to be used as the anode in organic light-emitting diodes. The J-V-L characteristics are shown in Fig. 7(a). The WF of the SLG/AgNWs (1.5 mg/ml) composite electrode was about 5.1 eV which is higher than ITO (~4.7 eV), making the hole injection easier. The turn-on voltage of the device with composite electrode was 2.5 V, which was similar to ITO-device, due to that the composite electrode had a higher WF. Figure 8 shows the energy level diagrams. The luminance intensity could reach 1000 cd m^−2^ at 6.0 V, and that of the ITO device reached the same intensity at about 4.0 V. This is because the lower current density than ITO reduced the carrier recombination at the same voltage. However, the current density of the composite electrode was considerably enhanced compared to graphene, the value increased from 55 mA cm^−2^ to 107 mA cm^−2^ at 9.5 V, which is consistent with the Hall-effect measurement that indicates that AgNWs successfully formed a conducting network to provide additional carriers for graphene. This consequently increased both current density and the luminance intensity. Figure 7(b) shows a schematic of the OLED structure. Figure 7(c) shows photographs of the flexible OLED with the SLG/AgNWs composite electrode.

## Discussion

In this work, we used AgNWs in conjunction with a graphene-based electrode to improve the common problem of poor connectivity and low carrier density of SLG. We studied the underlying mechanism of this improvement using percolation theory, and we determined the percolation concentration for this composite system. The SLG/AgNWs composite-electrode shows excellent photoelectric properties (Rs of 27 Ω/▯), high transmittance (86.7% at 550 nm), and it can bend over 1000 times with only small changes in conductivity. Flexible, organic light-emitting diodes, with 15000 cd m^−2^ luminance at 9 V driving voltage, were successfully fabricated with this composite electrode. In summary, we can confirm that combining AgNWs with SLG can not only effectively improve the conductivity of the graphene film but also meet the requirements to serve as effective transparent anode in flexible-OLED. These novel composite electrodes may open new possibilities for the next generation of efficient and flexible optoelectronic devices.

## Methods

### Preparation of the flexible SLG/AgNWs composite electrode

**Figure 9:**
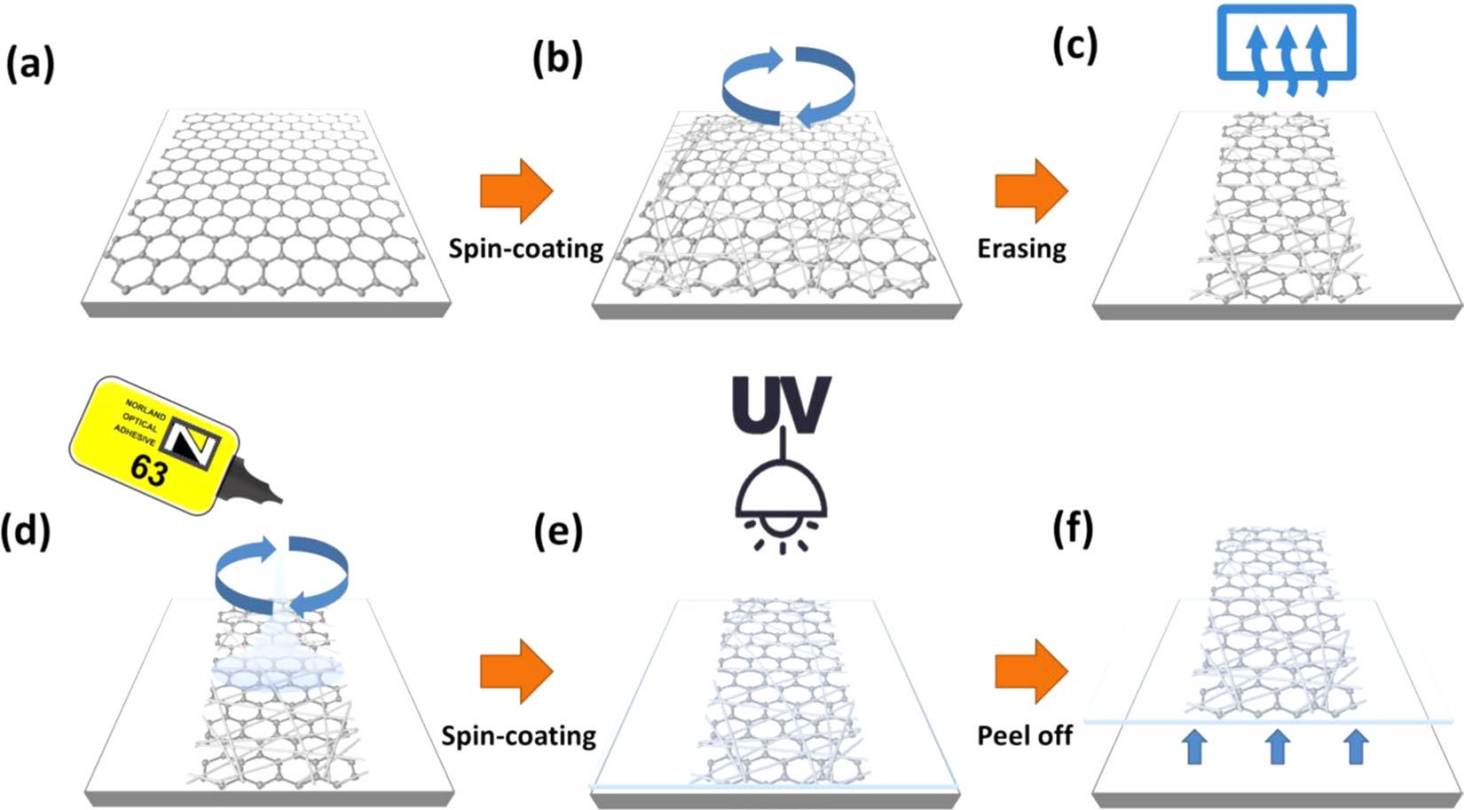
Flow-process of the fabrication of the flexible SLG/AgNWs composite electrode. (**a**) Deposited graphene on PET. (**b**) Spin-coating of AgNWs on Graphene-PET; (**c**) Drying the composite film at 150 °C for 10 min after patterning. (**d**) Spin-coating of the photoresist, NOA63, (**e**) Solidification of NOA63 under ultraviolet light for 4 min. (**f**) Peeling off of the composite film from the PET substrate to obtain the standalone flexible composite electrode.

**Figure 10:**
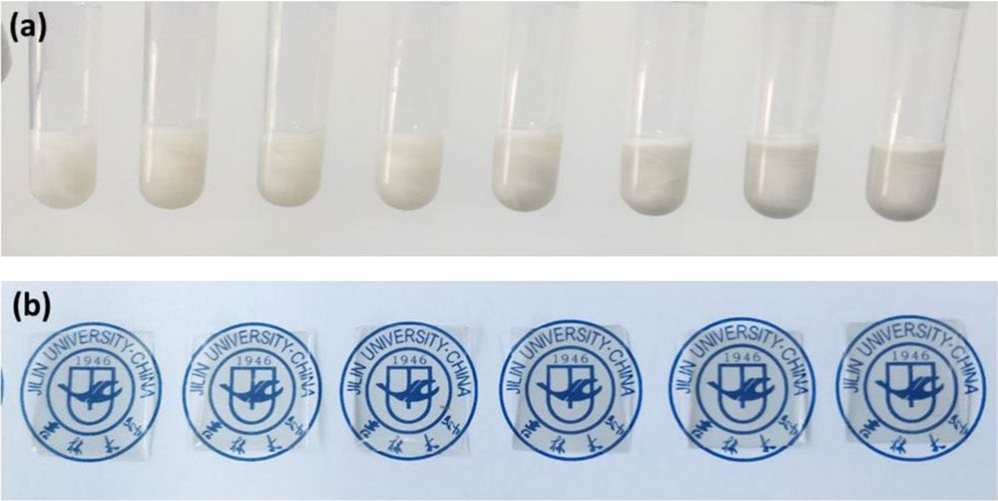
(**a**) Photographs of different concentrations of AgNWs (from left to right:0.1, 0.2, 0.3, 0.4, 0.5, 1.0, 1.5, 2.0 mg/mL). (**b**) The Photographs of SLG-AgNWs composite films with different concentrations of AgNWs (from left to right: 0.1, 0.3, 0.5, 1.0, 1.5, 2.0 mg/mL), even at high concentrations (2.0 mg/ml).

The graphene in our work was prepared using a CVD method that contains 95% single-layer rate and its sheet resistance is about 650 Ω/▯. The preparation process of composite electrode is shown in Fig. 9. The SLG was initially deposited on a PET substrate. We then spin-coated AgNWs with different concentration on SLG film at a spin speed of 3000 rpm - see Fig. 9b. The AgNWs solution with different concentrations is shown in Fig. 10(a). We removed the excess graphene on both sides to obtain the desired electrode shape and size, and then dried the SLG/AgNWs composite films in a vacuum oven at 150 °C for 10 min to remove any solvent on the AgNWs surface - see Fig. 9c. Then, we applied a photoresist, NOA63 (Norland Optics, USA), which can be solidified by UV irradiation, as the flexible substrate. This solves the problem that AgNWs are too rough to be used as electrodes on PET. NOA63 was dynamically spin-coated at 300 rpm for 15 s, followed by 600 rpm for 15 s. We then waited for two minutes to allow NOA63 to penetrate the gaps of AgNWs completely. After exposure to UV irradiation for 4 min (Fig. 9e), the photoresist solidified, and the smooth, transparent and flexible SLG/AgNWs composite film was removed from the PET substrate - see Fig. 9f. The physical diagrams of SLG-AgNWs composite films with different concentrations of AgNWs are shown in Fig. 10(b).

### Fabrication of OLED

To study its suitability as transparent electrode, we used this electrode to fabricate OLEDs. The structure of the OLEDs was the following: Anode (ITO or SLG/AgNWs composite electrode)/5 nm HAT-CN/40 nm TAPC/30 nm Alq_3_ doped with 1% C545T/30 nm Alq_3_/1 nm Liq and 100 nm Al.

### Characterization

Raman measurements were carried out using a Raman microscopy system equipped with a 532 nm wavelength laser. Optical transmittance was measured with a UV-Vis-IR spectrophotometer (UV3600, SHIMADZU). Sheet resistance was measured using a four-point probe. The charge-carrier concentration was measured with a Hall Effect Measurement System (HL5500PC). A self-built bending test system was used to assess the bending properties of the composite electrode. The current-density–voltage–luminance (J–V–L) characteristics of the flexible-OLED were simultaneously measured using a source-meter (Agilent B2902A) and a Minolta luminance-meter (LS-110). The work function (WF) was measured with a Kelvin probe system. The surface roughness was measured with an atomic force microscope (Bruker Dimension Icon-PT).

### Simulation by monte carlo method

The simulation process is as follows. The AgNWs are considered as long line segments. At the beginning, place the AgNWs with length value in normal distribution and negligible thickness on the plane randomly. Each nanowire stick is given a random azimuthal orientation (θ), from 0 to π. Both of them are generated by random functions. There are three parameter matrices which mean x, y, θ and an array of the numeral orders, we can call them IDs. For each recursion, we will set the nanowires in contact with each other to the same ID. At this point, the entire plane is not conductive. Then the total number of AgNWs will be increased by recursion until critical density. The critical density is defined as that the same ID could be found on both boundaries of plane, left and right, bottom or top. It means that the system will become a conductor, and the resistance will drop rapidly after that. In one word, through this simulation we acquire percolation threshold of the AgNWs system. The sheet resistances of different density were measured by experiment, to acquire more data to determine the relationship between them.
